# Proof of Concept of Scalable Integration of Internet of Things and Blockchain in Healthcare

**DOI:** 10.3390/s20051389

**Published:** 2020-03-03

**Authors:** Krishna Prasad Satamraju, Malarkodi B

**Affiliations:** Department of Electronics and Communication Engineering, National Institute of Technology, Tiruchirappalli, Tamil Nadu 620015, India; malark@nitt.edu

**Keywords:** blockchain, healthcare, Internet of Things, privacy, security, smart contracts

## Abstract

The advent of Internet of Things (IoT) brought innovation along with unprecedented benefits of convenience and efficacy in many operations that were otherwise very cumbersome. This innovation explosion has surfaced a new dimension of vulnerability and physical threat to the data integrity of IoT networks. Implementing conventional cryptographic algorithms on IoT devices is not future-proof as these devices are constrained in terms of computational power, performance, and memory. In this paper, we are proposing a novel framework, a unique model that integrates IoT networks with a blockchain to address potential privacy and security threats for data integrity. Smart contracts are instrumental in this integration process and they are used to handle device authentication, authorization and access-control, and data management. We further share a new design model for interfaces to integrate both platforms while highlighting its performance results over the existing models. With the incorporation of off-chain data storage into the framework, overall scalability of the system can be increased. Finally, our research concludes how the proposed framework can be fused virtually into any existing IoT applications with minimal modifications.

## 1. Introduction

With the advancements in technology, miniaturization due to modern VLSI (Very Large Scale Integration) related technologies, industries are evolving to meet the ever increasing needs of the society. Internet of Things, as coined by Kevin Ashton [1], has revolutionized the world with its potential to build cost-effective applications. The Internet of Things is meant for collaboration between networked devices that have built-in intelligence. IoT is a technology that has emerged from a combination of different technologies such as Machine-to-Machine communication, RFID, Supervisory Control and Data Acquisition (SCADA), and Wireless Sensor Networks. IoT provides a way to access and connect to devices that have unique identity. The dynamic, self-adaptive, and self-configurable nature of IoT network distinguishes them from the conventional wireless networks. 

**Figure 1:**
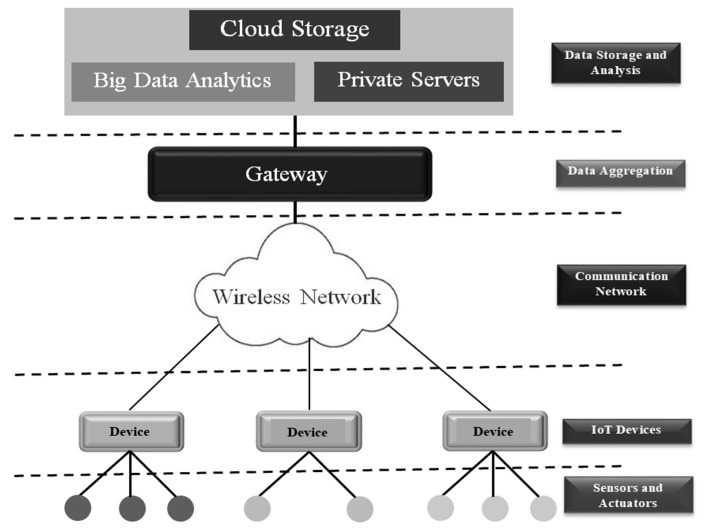
Generalized IoT structure.

The general IoT structure is shown in Figure 1. The layered architecture provides a variety of services at different levels which are essential for managing communication across different applications running over the entire network. These services include sensing data in the environment where the device is deployed, reacting to the commands based on the given criteria (ex. turning on a relay), processing the raw data, storing it for analysis purpose, communicating the data to a control node, and sharing of data with other applications. IoT has rapidly transformed the business facet with its broad spectrum of applications. The technology is instrumental in building applications such as smart cities, smart homes, smart agriculture, intelligent transport, and advanced medical and health applications [2,3,4,5,6,7,8].

Security in IoT is still a factor of concern and boundless applications of the IoT technology portend a wide range of security challenges [9,10]. Unauthorized access and data manipulation have become common threats in these networks. A survey [11] claims that, in the past three years, more than 20% of the organizations have suffered at least one IoT based attack. It also forecasts that the amount spent on the security of the IoT devices would increase to $3.1 billion dollars. IoT is a network of devices, constrained in terms of computational power and memory, and are battery power operated in the majority of the cases. For effective utilization of the energy by IoT devices, the authors of [12] proposed two powerful energy optimization techniques, namely, batching and Computation Off-loading to MCU (Micro Controller Unit). As the number of IoT devices grow across the world, the impact of these devices on the surrounding environment is of paramount importance. In [13], the authors proposed a three-step leverage free green energy solution that involves providing green energy harvesting, wireless green energy charging, and green energy balancing. 

The rest of the paper is organized as follows. Section 2 elaborates on the existing security mechanisms in the IoT networks and the blockchain, and elucidates the need for the integration of these two technologies. In Section 3, the proposed integration framework along with the key design issues are discussed. Section 4 evaluates the framework by considering a healthcare use case and discusses various functional aspects. Conclusions are presented in Section 5. 

## 2. Related Works

In general, any information system should comply with Confidentiality, Integrity, and Availability (CIA) requirements to guarantee security. Confidentiality refers to the protection of sensitive data from unauthorized access. Integrity guarantees that data are not modified by any unauthorized entity. Availability assures that data can be accessed at any time. In this section, various security mechanisms employed in IoT and blockchain are reviewed, and the need for integration of IoT and blockchain is explained.

### 2.1. Security Preliminaries

Let M be the message space containing all symbols defined by the alphabet (binary, English, or Hexadecimal) and m ∈ M be an element in a plain text message. Let C be the cipher text space consisting of symbols that are different from the one defined in M. The encryption mechanism is a bijective function, denoted by E_e_, between M and C, which can be uniquely determined by an element {e ∈ K}, where K is the key space. Likewise, the decryption mechanism that can be defined as a bijective function, denoted by D_d_, between C and M can be determined uniquely by {d ∈ K}. The encryption set is {E_e_ : e ∈ K} and decryption set is {D_d_ : d ∈ K}. The encryption and decryption mechanisms are defined as: ∀ e∈K ∃ d ∈ K, such that

(1) $$D_{d}{=\;E}_{e}^{-1}$$

where e and d are the encryption and decryption keys, respectively. Decryption of the message is given by

(2) $$D_{d}\left(E{\;}_{e}\left(m\right)\right)=m\;\forall \;m\in M$$

**Figure 2:**
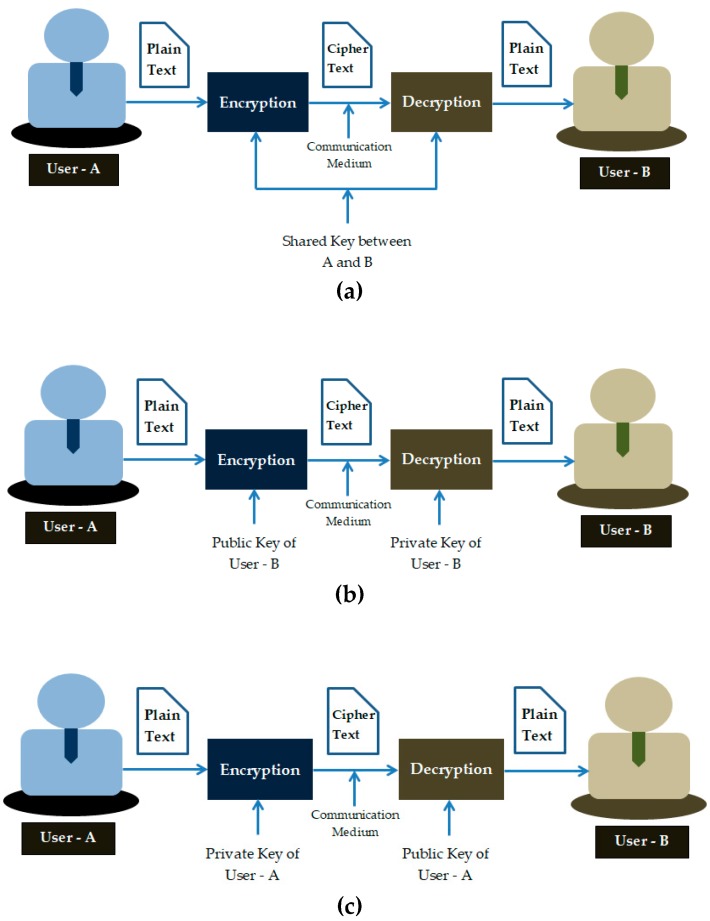
Cryptography types (**a**) symmetric cryptography; (**b**) asymmetric cryptography; (**c**) digital signature.

The {e, d} pair is known only to the communicating entities. If the key used for encryption and decryption process is the same (key is shared among the users involved in the communication), it is called symmetric cryptography. Asymmetric cryptography uses a public key and private (secret) key pair for encryption and decryption operations. Digital signatures use asymmetric cryptography inherently and provide integrity to the data. Figure 2 illustrates these cryptographic mechanisms. 

### 2.2. Security in IoT

Providing security in IoT networks is a complex process. Most of the security implementations are employed at server level and centralized security architecture is not feasible for the large scale IoT networks. Even though cryptographic functions used in the Internet can also be used in the IoT networks, better distributed and decentralized security mechanisms are needed to cope with the unmatched scaling levels. The lack of coordination among OEMs (Original Equipment Manufacturers) results in device heterogeneity and hence devising common security strategies is a challenge. The future IoT networks need versatile security solutions that provide scalable and distributed trust among its users.

An up-to-date review of IoT authentication protocols has been presented in [14]. The authors have evaluated and compared various protocols and provided a base for researchers and also highlighted various open issues and challenges that need to be addressed while developing new protocols in future. The authentication protocols for constrained devices should be lightweight, and there should be a trade-off between power consumption and security. 

Interactive and a non-interactive key management protocols have been proposed in [15]. The non-interactive version minimizes the communication cost in the IoT networks. This proposed scheme also enhances the security of traditional 4-way handshake models used in 802.11i and is resilient against many security attacks. 

A secured decision-making system based on inputs from different sources has been proposed in [16]. This system uses a number of virtual cells deployed in the edge infrastructure. This model enjoys the benefits derived from the edge computing but suffers when any one of the central components in the infrastructure fails. 

An end-to-end, SDN (Software Defined Radio) based framework for the evaluation of security in cloud based IoT networks has been proposed in [17]. Parameters such as physical device security, access control, authentication, data integrity, etc. are analyzed using a three-layer architecture in this work.

In [18], the authors presented a lightweight authentication protocol that ensures protection against known attacks in real-time RFID based IoT applications. The simulation results performed in this work supports both formal and informal security analysis at the expense of improved computing and communication cost.

Another novel model for providing security in the future generation IoT networks using security by contracts in Fog infrastructure has been proposed in [19]. This model envisages a feasible approach to create a trustworthy IoT environment using tractable contracts and policies. 

A comprehensive study on the security of an IoT based smart home has been presented in [20], where the authors have identified various security risks from both internal and external sources. This work mainly focused on identification of risks and their impact pertaining to the smart home application.

Physical Unclonable Function (PUF) is another technology that has potential to combat the security issues in IoT domain. They can provide a robust and lightweight solution for the computationally limited devices in the IoT networks compared to the current classical encryption techniques. A study in [21] provides a deeper understanding of the challenges and opportunities of using this technology for security in IoT domain. 

In [22], the authors proposed a lightweight framework for an IoT based healthcare system. This model can serve as a reference for building applications that use cost-effective and resource constrained devices. 

Digital certificates are extensively used in the traditional internet applications to provide data security and authentication. They consume a considerable amount of memory as well as computational power. However, in sophisticated IoT systems, these certificates can be hardcoded into the devices and when the device gets compromised, and there is an option for reloading the firmware. 

DER (Distinguished Encoding Rules) encoded X.509 certificates are widely accepted by open-source community for implementing authentication process and also support device interoperability. IoT application protocols such as MQTT use TLS (Transport Layer Security) for secured communication. MQTT is one of the most widely used protocols in the IoT domain and follows a publish/subscribe model operating over TCP/IP. It provides connection-oriented, ordered, and lossless bidirectional data transfer. A broker in MQTT enables the communication, processes the incoming packets, sorts them according to the topics, and forwards them to the subscribers of the corresponding topic. It has the lowest overhead and provides three Quality of Service (QoS) metrics for different levels of message reliability:

- QoS-0 is no guaranteed reliable level. There is no guarantee for the packet delivery.
- QoS-1 guarantees that the message is delivered at least once.
- QoS-2 guarantees that the message is received only once. This is safest but slow in service.

Services with QoS > 0 have better reliability with significantly increased delays. QoS-2 uses a four-way handshake and hence has higher delays compared to the other two. Hence, QoS-2 is not suitable for real-time application scenarios. Packet loss is a factor that affects the performance of the system employing MQTT protocols. Table 1 shows the impact of increased payload and number of users on the packet loss in MQTT QoS-0 and QoS-1 service. IoTIFY [23], a cloud-based performance testing platform for IoT based applications, has been used for simulations. It has built-in supports for cloud platforms such as Amazon Web Services (AWS) and Microsoft Azure. For the evaluation, an MQTT server connected by a link of 1000 Mbps shared bandwidth has been used with AWS as a cloud service. MQTT version 3.1.1 has been used for simulations. A system with core i7 8^th^ generation processor that runs on 2.2 GHz clock and 24 GB RAM has been used for simulating the results. 

From Table 1, it is clear that packet loss becomes significant in both the services as the payload and number of nodes increase. Critical applications such as healthcare and financial transactions require more reliable and sophisticated designs for trusted services. A minimal loss of data in such scenarios can create significant service interrupts. The TLS used for security in MQTT makes use of Public Key Infrastructure (PKI) for complex key generation, management, and distribution. Constrained IoT devices cannot accommodate such services. 

In short, most of the works discussed above lack in various aspects’ provisions of the overall security requirements of IoT networks. Hence, there is a need for a common platform that provides a single-point solution for all the security needs of the IoT networks. 

### 2.3. Blockchain Principles

Blockchain [24] is a decentralized public ledger which provides immutable, transparent, secure, and verifiable transactions over a distributed platform. It has the potential to solve the problem of providing trust for sensitive data flowing in information systems. The transactions in the blockchain are stored in a block after each transaction is verified by all the peers of the network using public key cryptography before being attached to the previous blocks. This process is called mining and the nodes that perform this mining are called miners. Each node in the chain maintains a copy of the entire transactional blocks. The consensus mechanism in blockchain is crucial for it to function correctly. The consensus mechanisms ensure that all the nodes in the blockchain are synchronized with each other, and all the transactions in the blocks are valid. Common consensus protocols are Proof of Work (PoW), Proof of Stake (PoS), Delegated PoS (DPoS), etc.

#### 2.3.1. Hash Functions and Merkle Trees

Hash function is another important component in building the blockchains. In a blockchain, the hash functions are used for mining or Proof of Work (PoW) and block generation. Merkle tree is a mathematical entity that is crafted as a tree of hashes of different blocks of data. This structure allows verification of arbitrary transaction in large volumes of data with a similar hash verification process used for a small amount of data. This method is very efficient and forms the core of the blockchain. The root hash indicates the hash of the entire data set.

**Figure 3:**
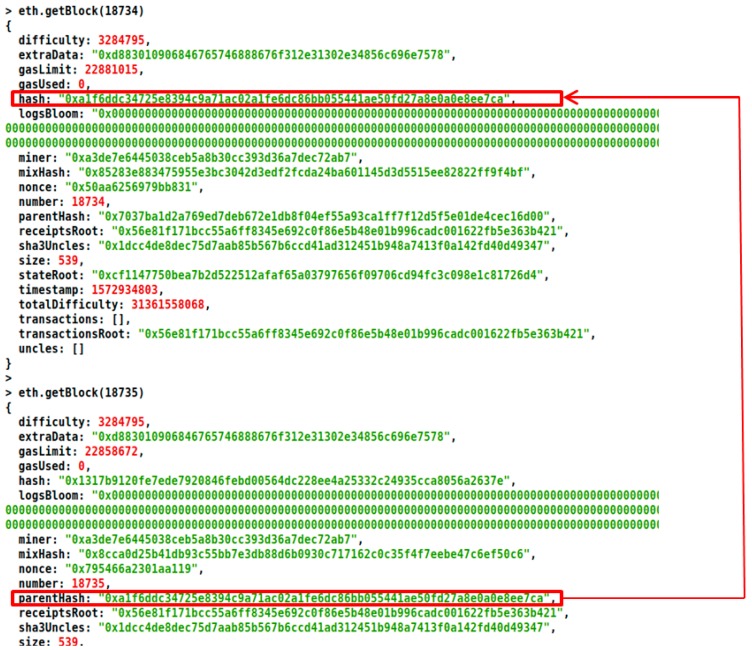
Merkle tree structure in a blockchain.

The Merkle tree structure between two successive blocks in a blockchain is shown in Figure 3. The Merkle tree is constructed in a bottom-up approach and is created by repeated calculation of hash pairs of the nodes until only one hash is left. Merkle tree is binary and hence will have an even number of leaves. 

Currently, researchers started exploiting the benefits derived by the incorporation of blockchain into various application scenarios. In [25], a privacy-aware PKI based system has been developed for permissioned blockchains. It proposes a digital certificate publishing scheme that helps in preserving the privacy of user identity and provides legitimate authorization.

Authors in [26] have provided a comparative analysis of available blockchain ecosystems. They analyzed various security and consensus mechanisms of different blockchain platforms and proposed key aspects required for the adaptation of the technology in the future applications. 

In [27], authors presented several security services available in the blockchain. Authors have presented a deep insight on the usage of these services for present day business requirements. The work highlights the security challenges in the existing systems and elucidates how blockchain can resolve these issues. 

A comprehensive review on various mining and consensus mechanisms in blockchain has been presented in [28]. This work emphasized the design of the permissionless consensus mechanisms and their applications in the broad area of telecommunication. 

Blockchain as a Service (BaaS) has been developed in [29] using a cloud infrastructure to provide blockchain services. This system supports network deployment and smart contract testing. This model also helps in developing business logic validations without the need for maintaining or monitoring the network. 

Another significant area where blockchain finds extensive application is in the logistics and supply chain management. In [30], authors presented a comprehensive review of various trends in applying blockchain based solutions in logistics and supply chain management. Blockchain has the potential to provide end-to-end traceability and authenticated ecosystem for the delivery of food products from the suppliers to end-users. 

The consensus mechanisms employed for transaction validations in the blockchain based models discussed above are computationally intensive. Considering the cost involved in the blockchain processing, these solutions are not viable for the majority of the IoT applications. Inclusion of smart contracts into blockchain has bought tremendous momentum to the blockchain based solutions revolutionizing the application scenarios where the blockchain can be used.

#### 2.3.2. Smart Contracts

A Smart contract is a piece of code that contains a set of terms governing the transactions over the blockchain network and executes these terms without any third-party intervention. A smart contract can be accessed by all its users using a contract’s address generated by the blockchain platform during its deployment stage. The combination of blockchain and smart contracts has revolutionized the current business scenarios and this combination is termed by developers as Blockchain 2.0. 

### 2.4. Need for the Blockchain and IoT Integration

Blockchain 2.0 based solutions can offer potentially solid solutions for various privacy and security issues in IoT. The inclusion of smart contracts into blockchain has made it possible to unleash different possibilities in applying trustless decentralization. The decentralized and autonomous nature of the blockchain makes it best suitable for integrating with IoT networks. Moreover, this integration can provide solutions to the challenges emphasized in [31], such as,

- **Decentralization:** Centralized systems such as cloud based IoT networks are single points of failures. Blockchain provides decentralized peer-to-peer architecture.
- **Scalability:** Scalability is the outcome of decentralization which improves fault tolerance.
- **Identity:** Every IoT device connected to the blockchain can be addressed uniquely and blockchain can provide distributed authorization and authentication to these devices.
- **Autonomy:** The IoT devices can interact with each other using the blockchain infrastructure without the need for centralized servers.
- **Reliability:** Tamper-proof and distributed record management feature of the blockchain can bring a higher degree of reliability for the data from the IoT devices.
- **Security:** The information from the IoT devices is stored as transactions inside the blockchain. Before storing the data inside blocks, each transaction is validated by all its peers inside the blockchain. In this way, security is guaranteed.

Few applications that are developed based on the combination of IoT and blockchain smart contracts are listed in Table 2.

By using technologies such as LoRa (Long Range), a spread spectrum modulation technique derived from chirp spread spectrum (CSS) technology, Swarm, a distributed data storage platform, and Ethereum [41], new paradigms for IoT backend with a combination of Distributed Denial of Service (DDoS) resistant and fault tolerant storage systems can be developed [42]. Blockchain based IoT applications require strong understanding of complex scenarios, and relevant architectural and logical support. There are many challenges yet to be addressed with this integration process [43]. In [44], authors have furnished a systematic analysis of relevant work on this ground.

In the next section, the proposed framework is presented. The components of the framework, their interactions, and key design issues are elaborated.

## 3. Proposed Framework

**Figure 4:**
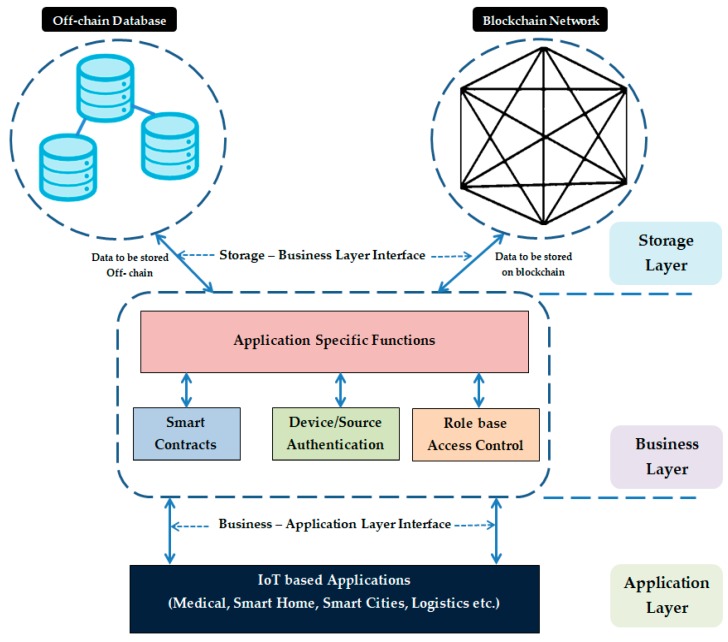
Proposed framework for IoT and blockchain integration.

Figure 4 shows the overall structure of the proposed framework. It is a three-layer structure that accommodates all functionalities required for the integration process. 

### 3.1. Application Layer

This layer provides an interface between the IoT devices and the blockchain services. The legitimate IoT devices and other system users can access the system services such as database storage, access control, and communication between other user applications. The users in the proposed model can be physical IoT devices with sensors installed or any Decentralized Application (DApp) browsers such as Metamask [45]. These browsers are usually provided with developer tools, preferably a Graphical User Interface, which helps programmers to develop application-specific functions. Each user of the system will be assigned a set of roles based on which they get services from the business layer. 

### 3.2. Business Layer

This layer is the core of framework and contains all the logic required to run different applications in the system. It acts as an abstraction layer between the IoT applications and the blockchain. The functions specified in this layer are reusable and are coded purely based on the application requirements. Services such as smart contracts, user validations, access control, etc. reside in this layer. 

### 3.3. Storage Layer 

**Figure 5:**
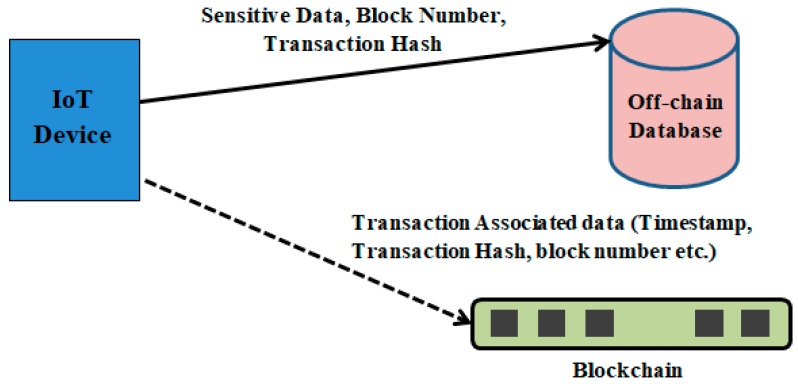
Off-chain based data storage mechanism.

Data privacy is a major constraint for sensitive data stored in the network. To increase privacy, the data stored in the blockchain are encrypted [46]. However, data encrypted by the users are visible to all the peers in the network. Therefore, in order to protect sensitive data, the proposed model uses off-chain data storage. The sensitive data are stored in a private database, and the blockchain stores associate information required for validating the integrity of data with a timestamp. This method of storing data is usually termed as off-chain storing. Figure 5 illustrates this process. Storing of associate data in the blockchain also allows verification of immutability.

By avoiding bottlenecks and single points of failure, the system can be designed to be fault-tolerant. The off-chain mechanism is best suited for IoT applications because storing such large volumes of data over the chain is expensive. The business layer contains dedicated control mechanisms to ensure that the off-chain database can only be accessed by authorized entities. 

In the next section, the performance of the proposed framework is evaluated by considering a healthcare system. Then, the components of the system, software, hardware, and other issues related to the system are described in detail. 

## 4. System Evaluation

**Figure 6:**
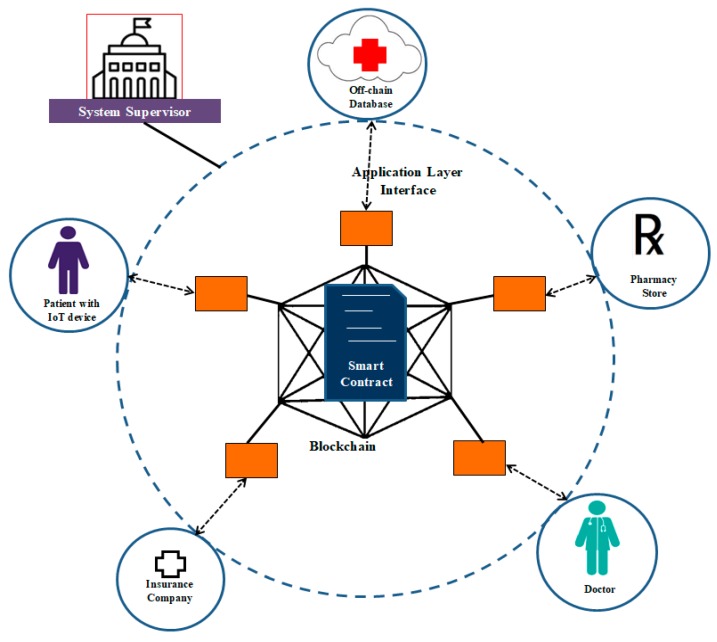
Overview of the use case scenario.

In order to evaluate the performance of the proposed framework, a healthcare use case has been developed using the features mentioned in the proposed framework. Figure 6 shows the overall scenario of the healthcare system. This system uses an Ethereum based permissioned blockchain. The permissioned blockchain is the one that allows only known nodes into the network which are given complete authority to validate the transaction blocks. Various nodes in the system are described below:

- **IoT Device:** This device is attached to the patient body to monitor vital parameters such as heart rate and body temperature. Sensors that read these parameters from the body are attached to the controller in the device. Processing of raw data received from the sensors, framing the data for storage purposes, communication, and networking functions will be taken care of by this device. All the interactions will go through the blockchain network, and each of these transactions are stored as immutable records inside the network. The sensitive data from the device are stored in the off-chain database.
- **Off-Chain Database:** This is the database in which the body vital parameters and other patient records are stored. Access to this database is controlled by the smart contract. Read or write operations on this database are based on the privileges assigned to the users by the system supervisor. There are security mechanisms employed at the database level to accommodate data privacy and integrity. Optionally, data can be hashed before being stored in the database.
- **Doctor:** A doctor can use a DApp to access the database to monitor the concerned patient’s body vital parameters and prescribe medicines based on the observations. Only authorized doctors are allowed to view data of a particular patient or to prescribe medication.
- **Pharmacy:** The pharmacist, using a DApp, can access medical prescription of a particular patient upon proving his identity. He can also access the address of the patient so that the medicines can be directly delivered at the patient’s site.
- **Insurance Company:** The insurance company is another component in this system who can access the services using a DApp. When a claim is made by the patient, he has the authority to verify the patient records.

The advantages of this system are:

1. It provides real-time monitoring of patient’s critical conditions.
2. It provides security to the sensitive data of the patient.
3. It helps in making the insurance claiming process transparent as the records inside the blockchain are immutable and provide end-to-end traceability.

### 4.1. Implementation Details

#### 4.1.1. System Supervisor

System supervisor is the one who resides in the hospital management and is responsible for assigning privileges and different access permissions to the system users. There are four nodes in this system, and the privileges for each node are listed in Table 3. The smart contract deployment is also the responsibility of the supervisor. 

#### 4.1.2. Software

Among the available blockchain development platforms, the most popular are Ethereum, Hyperledger [47], and IBM Blockchain [48]. For the evaluation of this model, Ethereum is being used as a platform. The software packages and libraries used in the design are listed in Table 4. 

An Ethereum node is created by using geth. geth is the implementation of Ethereum node in Go language. Truffle [49], an Ethereum based development and testing framework that is built over the Ethereum Virtual Machine (EVM), has been used for generating executable byte code. Truffle has in-built support for smart contracts’ compilation and linking. The environment of the Truffle framework also supports binary management and smart contract deployment. It also supports automated contract testing for rapid prototyping of applications. In order to reduce the computational costs and complexity, this model uses lightweight smart contracts instead of conventional consensus mechanisms [50] to record transactions and allow access to the resources.

Solidity, the official programming language to build smart contracts in Ethereum based blockchains, is used to code the smart contracts. Web3.py, a Python API based on web3.js, provides interaction between applications and the smart contracts. Smart contracts play a vital role in the proposed integration model. The functions provided by the smart contract interface and their description are given in Table 5. 

The system assumes that all the users have a private key and public keys of all the entities which are involved in communication. 

The APIs to interact with smart contracts are written using Python. Peer connectivity between the heterogeneous nodes (miner and IoT devices) poses a challenge in the system design. The *enode* information about a particular node can be used to add peers to the miners. Algorithms 1–5 for various operations performed by the user are given below.
**Algorithm 1: Storing patient body vital parameters in the database**Input: patientID, patient_body_parameters Output: Body parameters are stored in the database and Transaction is recorded. pragma solidity ^0.5.12; mapping(address => bool) authorizedPatients; if( isPatientAuthorized(patientID))   *store the patient body parameters in corresponding patient’s record;*  *transaction is recorded in the blockchain;*  *store the transaction hash and block number in the patient record;* } else  *Revert the transaction;*   function public isPatientAuthorized(address patientID) public view return (bool approved) {    return authorizedPatients[patientID];  }
**Algorithm 2: Monitoring patient body vital parameters**Input: patientID, doctorID Output: patient_body_parameters pragma solidity ^0.5.12; mapping(address => address) authorizedDoctor; if( isDoctorAuthorized(patientID)) {  *Read the patient body parameters;*
  *transaction is recorded in the blockchain;* } else *Revert the transaction;* function public isDoctorAuthorized(address patientID) public view return (bool display) {   if(msg.sender == authorizedDoctor(patientID))    return true;   else     return false;  }
**Algorithm 3: Update patient prescription**Input: patientID, doctorID, prescription Output: Patient record update with new prescription pragma solidity ^0.5.12; mapping(address => address) authorizedDoctor; if( isDoctorAuthorized(patientID))  {  *Update the prescription in the corresponding patient’s record;*  *transaction is recorded in the blockchain;* } else  *Revert the transaction;* function public isDoctorAuthorized(address patientID) public view return (bool display)  {   if(msg.sender == authorizedDoctor(patientID))    return true;   else     return false; }
**Algorithm 4: Accessing patient’s prescription**Input: patientID, pharmaID, Output: Prescription of the patient pragma solidity ^0.5.12; mapping(address => address) authorizedPharma; if( isPharmacyAuthorized(patientID)) {  *Get prescription of the patient from the database*  *transaction is recorded in the blockchain;* } else  *Revert the transaction;* function public isPharmacyAuthorized(address patientID) public view return (bool display) {   if(msg.sender == authorizedPharma(patientID))    return true;   else     return false; }
**Algorithm 5: Accessing patient’s medical records by the insurer**Input: patientID, insurerID, Output: Records of the patient pragma solidity ^0.5.12; mapping(address => address) authorizedInsurer; if( isInsurerAuthorized(patientID)) {  *Get patient’s record from the database;*  *transaction is recorded in the blockchain;* } else  *Revert the transaction;* function public isInsurerAuthorized(address patientID) public view return (bool display) {   if(msg.sender == authorizedInsurer(patientID))    return true;   else     return false; }

#### 4.1.3. Hardware 

In this use case, we have devised two medical IoT devices, one using Raspberry Pi 3 Model B [51] and the other using Raspberry Pi 3 Model B+ [52], to collect body vital parameters from the patient’s body and to store in the off-chain database after proper authentication. Application frameworks such as [53] also used Raspberry Pi based mini computers to successfully evaluate the performance of IoT based logistics test bed. The MAX30100 Pulse Oximeter sensor and MAX30205 body temperature sensor and Pmod TMP3 temperature sensor from Digilent Inc. are used to measure heart rate, body temperature, and room temperature, respectively. Both of the devices are loaded with the necessary libraries and packages to accomplish the job of a blockchain node and carry out the transactions with other nodes in the network. 

**Figure 7:**
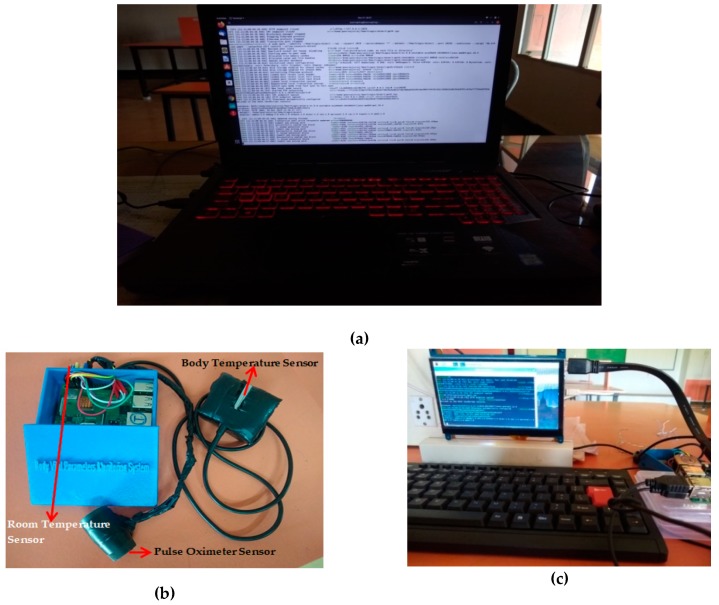
Hardware Setup (**a**) miner; (**b**) medical IoT Device-1 (Raspberry Pi 3 Model B+); (**c**) medical IoT Device-2 (Raspberry Pi 3 Model B).

The hardware setup is shown in Figure 7. The system contains one miner (Figure 7(a)), which is a high end computing system running on an Intel Core i7 8^th^ Generation processor, 2.2 GHz clock frequency, 24 GB RAM, and 1 TB secondary storage. The operating system used is Ubuntu 18.04. The two IoT devices using Raspberry Pi boards are shown in Figure 7b,c.

### 4.2. Analysis

In the proposed model, each user is assigned with a restricted level of privileges. Access to the database is provided only to the authorized users. The framework provides a two-tier security for the resource access with a blockchain self-security mechanism at the first stage and smart contract based access control at the second stage. 

Table 6 lists the average time taken by different processes in the proposed model. Various parameters related to the blockchain process are detailed below.

- **Transaction Confirmation time:** A node in the chain is expected to validate each transaction of every block. Hence, transaction validation is one of the major tasks in the blockchain. The computational power of the system has an unswerving effect on the transaction confirmation time. The average transaction confirmation time of the system is 1.7 seconds.
- **Block Time:** The block time is defined as the amount of time it takes for the miner to generate a new block. In Ethereum, the block time is between 10–20 seconds. In the proposed use case, it is 11.21 seconds.
- **Migration and Deployment Time:** It is the amount of time taken by the smart contract testing framework (Truffle in this case) to compile the smart contract and push it on to the Ethereum network. It took 9.14 seconds to deploy the smart contract in this use case.
- **Deployment Cost:** It is the fee paid by the user to push the smart contract application on the Ethereum blockchain. The deployment cost in the proposed model is 0.00179117 ETH.

**Figure 8:**
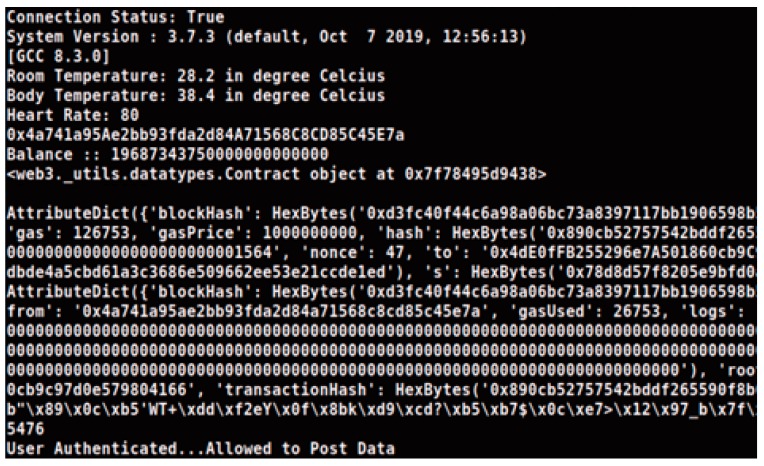
Result demonstrating successful storing of patient body vital parameters.

Figure 8 demonstrates the result of storage of the body vital parameters in the database after successful authentication. SHA-3 256 has been used to store the transactional hashes inside the blocks. 

The proposed framework is compared with the relative works in the same domain, and the comparative analysis is summarized in Table 7. 

#### 4.2.1. Discussion

The proposed model is successfully implemented over an Ethereum based permissioned blockchain. It is evident that, even though the integration of IoT devices with blockchain is complex, the in-built features such as transaction validations by all the peers and immutability of the transactions inside the blocks make this option a better choice for securing the IoT applications. Any alteration or device compromise can be easily identified by the other nodes in the network. This framework also offers a better security solution for IoT devices compared to the traditional security services offered by PKI systems. The two key challenges, namely confidentiality and scalability, are addressed with the off-chain solution. The advantages derived by the IoT applications from this integration process are discussed below:

- **Scalability:** Scalability is defined as the ability of an information system to maintain its equilibrium state with increased storage volume. Scalability is a key issue in the integration of blockchain and IoT as IoT devices are growing rapidly and their applications, in general, generate huge volumes of data. In [50,54], the scalability is achieved at the cost of increased complexity due to the clustering of nodes, and lifetime management of these clusters. In addition, the method described in [54] requires each node to store at least one local blockchain at any instance of time and hence it is not suitable for memory-constrained IoT devices. Furthermore, with off-chain data storage mechanisms, only associated data are stored in blockchain, and sensitive data are stored in the off-chain database. This reduces the transaction data size and increases the number of transactions that can be accommodated within the block. Hence, throughput and scalability of the overall system are enhanced.
- **Confidentiality:** As the proposed model uses permissioned blockchain, only authorized users are allowed to access the blockchain network. Since only authorized users can access on-chain as well as off-chain data, the confidentiality of the data is preserved.
- **Access control and tamper-proof:** Role-based access to the database is enabled by the smart contract that is deployed on the blockchain platform. The tamperproof nature of the blockchain makes it even more difficult for someone to modify the transaction data on chain.

The novelty of the proposed framework is derived from the fact that it creates an ecosystem wherein traditional IoT devices having insecure data transfer, storage constraints, and insufficient privacy mechanisms can function seamlessly in a decentralized distributed and trustworthy system. The off-chain mechanism allows a means to relocate the storage and computational processes without compromising the inherent features of the blockchain technology. This feature is especially useful to combat the expensive storage and processing charges when this permissioned chain is connected to the Ethereum main chain.

## 5. Conclusions

The growth rate of IoT devices is tremendous, and there is always a need for the development of improved and efficient protocols to meet the required standards in terms of data privacy and security in IoT networks. Limitations such as scalability, latency, and packet loss are the major hurdles in the conventional IoT security protocols. A framework that uses integration of IoT and blockchain has been proposed in this paper to address these issues. With this integration model, the IoT applications can now use the inherent features of blockchain such as immutable record keeping as well as end-to-end traceability. A proof of concept has been developed based on the healthcare system for evaluating the performance of this framework. The system has been implemented on a permissioned Ethereum blockchain platform which supports smart contracts. Four different users with different privilege levels of access have been considered. The performance of this system is compared with similar models in terms of access control, scalability, and confidentiality to highlight the significance of the proposed model. This framework serves as a single-point solution for all the security needs of resource-constrained IoT networks. The framework provides a cost effective solution for many real-time applications where security is pivotal. More matured and value-added solutions can be built over the blockchain to further enhance the scalability and security of IoT applications in the future. This work can be further broadened to encompass social network applications so as to make blockchain inclusive in order to derive the benefits of both the applications resulting in secure social platforms. 

## Figures and Tables

**Table 1:** Packet loss in MQTT QoS-0 and QoS-1 services with variable loads and users.

Payload Size	No. of Nodes	Type of Service	Average Latency (mSec)	% of Packets Lost
100 bytes	100	QoS-0	0.017	0
		QoS-1	670.713	0
	200	QoS-0	0.016	0
		QoS-1	2029.758	0
	500	QoS-0	0.019	0
		QoS-1	6410.813	0
	750	QoS-0	0.019	0
		QoS-1	11592.543	1
	1000	QoS-0	0.017	0
		QoS-1	14771.078	13
1 KB	100	QoS-0	0.029	0
		QoS-1	735.773	0
	200	QoS-0	0.017	0
		QoS-1	2180.992	0
	500	QoS-0	0.017	0
		QoS-1	5144.808	1
	750	QoS-0	0.017	19
		QoS-1	7372.016	1
	1000	QoS-0	0.019	0
		QoS-1	13362.244	27
10 KB	100	QoS-0	0.017	0
		QoS-1	687.842	0
	200	QoS-0	0.017	0
		QoS-1	1746.986	0
	500	QoS-0	0.016	7
		QoS-1	5490.489	1
	750	QoS-0	0.016	1
		QoS-1	4649.574	1
	1000	QoS-0	0.016	0
		QoS-1	7923.592	8

**Table 2:** Applications of a smart contract based blockchain and IoT.

Reference No.	Category	Platform	Challenge Addressed
[32]	Industrial IoT	Multiplatform	Privacy, Security
[33]	Smart Transportation	Ethereum	Authentication, Privacy
[34]	Wireless IoT Systems	Ethereum	Security, Authentication
[35]	Smart Cities	Ethereum	Security
[36]	Internet of Things	Ethereum	Access Control
[37]	Electric Vehicle Charging	Multiplatform	Access Control
[38]	Smart Meters	Ethereum	Access Control
[39]	Asset Management	Multiplatform	Access Control
[40]	Blockchain based IoT	Multiplatform	Identity, security

**Table 3:** Privileges assigned to the users of the system.

Privilege ID	User	Privileges
*1*	Patient	Can store body vital parameters in the off-chain server and can read doctor’s prescription.
*2*	Doctor	Can access patient’s data and can update prescription.
*3*	Pharmacist	Can access doctor’s prescription and address of the patient from the database.
*4*	Insurance Company	Can access patient data, prescriptions and other records during claim validations

**Table 4:** Packages and Libraries used in the system design.

Package / Library	Version
geth	1.9.6
ethereum	1.0.8
eth_abi	2.0.0
ethjsonrpc	0.3.0
Truffle	5.0.39
Solidity	0.5.12
Py_solc	3.2.0
Node	10.15.2
Web3.js	1.2.1
Python	3.7.3
go	1.10.4

**Table 5:** Functions used in smart contracts and their description.

Function	Input Parameters	Return Value
isPatientAuthorized()	patientID	**true:** if the patientID is legitimate **false:** if the patientID is not legitimate
isDoctorAuthorized()	patientID	**true:** if the doctorID is authorized to view the corresponding patient data. **false:** otherwise
isPharmacyAuthorized()	patientID	**true:** if the pharmacyID is authorized to view the corresponding patient data. **false:** otherwise
isInsurerAuthorized()	patientID	**true:** if the insurerID is authorized to view the corresponding patient records. **false:** otherwise
storePatientInfo()	patientID, patient_body_parameters	Patient’s data is stored in the database after successful authentication of the patient—else the transaction is reverted and function reports FAILED transaction.
monitorPatient()	patientID, doctorID	Patient’s data is retrieved and from the database after successful authentication of the doctor—else the transaction is reverted and function reports FAILED transaction.
prescribeMedicines()	patientID, doctorID, prescription	Prescription is stored in the database after successful authentication process—else the transaction is reverted and function reports FAILED transaction.
getPrescription()	patientID, pharmacyID	Retrieves prescription of the patient from the database after successful authentication of the pharmacy—else the transaction is reverted and function reports FAILED transaction.
getPatientRecord()	patientID, insurerID	Patient’s records are retrieved from the database after successful authentication of the insurance company—else the transaction is reverted and function reports FAILED transaction.

**Table 6:** Average time taken by different processes in the system.

Process	Time
SHA-3 256	193 μS on Raspberry Pi 3 B 191 μS on Raspberry Pi 3 B+
Block time	11.21 Seconds
Migration and deployment of smart contract	9.14 Seconds
Transaction confirmation time	1.7 Seconds
Total deployment cost	0.00179117 ETH

**Table 7:** Comparative analysis of proposed framework with related works.

Method	Blockchain Platform	Authentication	Access Control	Authorization	Scalability	Smart Contract	Off-Chain Storage
[54]	Bitcoin	**✓**	**✓**	**✓**	**✓**	**✕**	**✕**
[55]	Multi Platform	**✓**	**✓**	**✓**	**✕**	**✕**	**✕**
[56]	Hyperledger Fabric	**✓**	**✓**	**✓**	**✕**	**✓**	**✕**
[57]	Ethereum	**✓**	**✓**	**✓**	**✕**	**✕**	**✕**
[58]	Multi Platform	**✓**	**✓**	**✓**	**✓**	**✕**	**✕**
Proposed Method	Ethereum	**✓**	**✓**	**✓**	**✓**	**✓**	**✓**

## References

[1] Ashton K. (2009). That Internet of Things Thing. RFID J..

[2] Rajab H., Cinkel T. IoT based Smart Cities. Proceedings of the International Symposium on Networks, Computers and Communications (ISNCC).

[3] Miguez F., Fernandez-Caranes T.M., Fraga-Lamas P., Castedo L. (2018). Design, Implementation and Practical Evaluation of an IoT Home Automation System for Fog Computing Applications Based on MQTT and Zigbee-WiFi Sensor Nodes. Sensors.

[4] Verma H., Jain M., Goel K., Vikram A., Verma G. Smart home system based on Internet of Things. Proceedings of the 3rd International Conference on Computing for Sustainable Global Development (INDIACom).

[5] Li W., Logenthiran T., Phan V., Woo W.L. (2019). A Novel Smart Energy Theft System (SETS) for IoT-Based Smart Home. IEEE Internet Things J..

[6] Satamraju K.P., Shaik K., Vellanki N. (2017). RURAL BRIDGE: A novel system for smart and co-operative farming using IoT architecture. Proceedings of the 2017 International Conference on Multimedia, Signal Processing and Communication Technologies (IMPACT).

[7] Agale R.R., Gaikwad D.P. Automated Irrigation and Crop Security System in Agriculture Using Internet of Things. Proceedings of the 2017 International Conference on Computing, Communication, Control and Automation (ICCUBEA).

[8] Herrera-Quintero L.F., Vega-Alfonso J.C., Banse K.B.A., Carrillo Zambrano E. (2018). Smart ITS Sensor for the Transportation Planning Based on IoT Approaches Using Serverless and Microservices Architecture. IEEE Intelligent Transp. Sys. Magazine..

[9] Neshenko N., Bou-Harb E., Crichigno J., Kaddoum G., Ghani N. (2019). Demystifying IoT Security: An Exhaustive Survey on IoT Vulnerabilities and a First Empirical Look on Internet-Scale IoT Exploitations. IEEE Comm. Surveys & Tutorials..

[10] Hassija V., Chamola V., Saxena V., Jain D., Goyal P., Sikdar B. (2019). A Survey on IoT Security: Application Areas, Security Threats, and Solution Architectures. IEEE Access.

[11] (2018). Forecast: IoT Security, Worldwide. https://www.gartner.com/en/newsroom/press-releases/2018-03-21-gartner-says-worldwide-iot-security-spending-will-reach-1-point-5-billion-in-2018.

[12] Zhao S., Rengasamy P.V., Zhang H., Bhuyan S., Nachiappan N.C., Sivasubramaniam A., Kandemir M.T., Das C. Understanding Energy Efficiency in IoT App Executions. Proceedings of the 2019 IEEE 39th International Conference on Distributed Computing Systems (ICDCS).

[13] Liu X., Ansari N. (2019). Toward Green IoT: Energy Solutions and Key Challenges. IEEE Comms. Magazine.

[14] El-hajj M., Fadlallah A., Chamoun M., Serhrouchni A. (2019). A Survey of Internet of Things (IoT) Authentication Schemes. Sensors.

[15] Celia L., Cungang Y. (WIP) Authenticated Key Management Protocols for Internet of Things. Proceedings of the 2018 IEEE International Congress on Internet of Things (ICIOT).

[16] Roman R., Rios R., Onieva J.A., Lopez J. (2019). Immune System for the Internet of Things Using Edge Technologies. IEEE Internet Things J..

[17] Han Z., Li X., Huang K., Feng Z. (2018). A Software Defined Network-Based Security Assessment Framework for CloudIoT. IEEE Internet Things J..

[18] Mansoor K., Ghani A., Chaudhry S.A., Shamshirband S., Ghayyur S.A.K. (2019). Securing IoT-Based RFID Systems: A Robust Authentication Protocol Using Symmetric Cryptography. Sensors.

[19] Giaretta A., Dragoni N., Massacci F. (2019). IoT Security Configurability with Security-by-Contract. Sensors.

[20] Ali B., Awad A.I. (2018). Cyber and Physical Security Vulnerability Assessment for IoT-Based Smart Homes. Sensors.

[21] Babaei A., Schiele G. (2019). Physical Unclonable Functions in the Internet of Things: State of the Art and Open Challenges. Sensors.

[22] Satamraju K.P., Malarkodi B. Design and Evaluation of a Lightweight Security Framework for IoT Applications. Proceedings of the TENCON 2019–2019 IEEE Region 10 Conference (TENCON).

[23] IoTIFY, a cloud based IoT performance testing platform. https://iotify.io/.

[24] Nakamoto S. (2008). Bitcoin: A Peer-to-Peer Electronic Cash System. https://bitcoin.org/bitcoin.pdf.

[25] Wang R., He J., Liu C., Li Q., Tsai W., Deng E. A Privacy-Aware PKI System Based on Permissioned Blockchains. Proceedings of the 2018 IEEE 9th International Conference on Software Engineering and Service Science (ICSESS).

[26] Ali Syed T., Alzahrani A., Jan S., Siddiqui M.S., Nadeem A., Alghamdi T. (2019). A Comparative Analysis of Blockchain Architecture and its Applications: Problems and Recommendations. IEEE Access.

[27] Salman T., Zolanvari M., Erbad A., Jain R., Samaka M. (2019). Security Services Using Blockchains: A State of the Art Survey. IEEE Comm. Surveys Tutorials.

[28] Wang W., Hoang D.T., Hu P., Xiong Z., Niyato D., Wang P., Wen Y., Kim D.I. (2019). A Survey on Consensus Mechanisms and Mining Strategy Management in Blockchain Networks. IEEE Access.

[29] Zheng W., Zheng Z., Chen X., Dai K., Li P., Chen R. (2019). NutBaaS: A Blockchain-as-a-Service Platform. IEEE Access.

[30] Tijan E., Aksentijević S., Ivanić K., Jardas M. (2019). Blockchain Technology Implementation in Logistics. Sustainability.

[31] Al-Fuqaha A., Guizani M., Mohammadi M., Aledhari M., Ayyash M. (2015). Internet of Things: A Survey on Enabling Technologies, Protocols, and Applications. IEEE Comm. Surveys & Tutorials.

[32] Wan J., Li J., Imran M., Li D., Fazal-e-Amin (2019). A Blockchain-Based Solution for Enhancing Security and Privacy in Smart Factory. IEEE Trans. Ind. Inform..

[33] Zheng D., Jing C., Guo R., Gao S., Wang L. (2019). A Traceable Blockchain-Based Access Authentication System With Privacy Preservation in VANETs. IEEE Access.

[34] Sun Y., Zhang L., Feng G., Yang B., Cao B., Imran M.A. (2019). Blockchain-Enabled Wireless Internet of Things: Performance Analysis and Optimal Communication Node Deployment. IEEE Internet Things J..

[35] Lindsay J. Smart Contracts for Incentivizing Sensor Based Mobile Smart City Applications. Proceedings of the 2018 IEEE International Smart Cities Conference (ISC2).

[36] Zhang Y., Kasahara S., Shen Y., Jiang X., Wan J. (2019). Smart Contract-Based Access Control for the Internet of Things. IEEE Internet Things J..

[37] Zhang K., Mao Y., Leng S., He Y., Maharajan S., Gjessing S., Zhang Y., Tsang D.H.K. (2018). Optimal Charging Schemes for Electric Vehicles in Smart Grid: A Contract Theoretic Approach. IEEE Trans. Intell. Transp. Sys..

[38] Thomas L., Long C., Burnap P., Wu J., Jenkins N. (2017). Automation of the supplier role in the GB power system using blockchain-based smart contracts. CIRED-Open Access Proc. J..

[39] Cruz J.P., Kaji Y., Yanai N. (2018). RBAC-SC: Role-Based Access Control Using Smart Contract. IEEE Access.

[40] (2017). Chain of Things. https://www.blockchainofthings.com/.

[41] Ethereum: a global, open-source platform for decentralized applications. https://ethereum.org/.

[42] Ozyilmaz K.R., Yurdakul A. (2019). Designing a Blockchain-Based IoT with Ethereum, Swarm, and LoRa: The Software Solution to Create High Availability with Minimal Security Risks. IEEE Consumer Elec. Mag..

[43] Ali M.S., Vecchio M., Antonelli F. (2018). Enabling a Blockchain-Based IoT Edge. IEEE Internet Things Mag..

[44] Lo S.K., Liu Y., Chia S.Y., Xu X., Lu Q., Zhu L., Ning H. (2019). Analysis of Blockchain Solutions for IoT: A Systematic Literature Review. IEEE Access.

[45] Metamask - Brings Ethereum to your browser. https://metamask.io/.

[46] Kosba A., Miller A., Shi E., Wen Z., Papamanthou C. Hawk: the blockchain model of cryptography and privacy-preserving smart contracts. Proceedings of the 2016 IEEE Symposium on Security and Privacy (SP).

[47] Hyperledger: Open Source blockchain Technologies. https://www.hyperledger.org/.

[48] IBM Enterprise Blockchain Solutions and Services. https://www.ibm.com/in-en/blockchain.

[49] Truffle Suite: Sweet Suite for Smart Contracts. https://www.trufflesuite.com.

[50] Bach L.M., Mihaljevic B., Zagar M. Comparative analysis of blockchain consensus algorithms. Proceedings of the 2018 41st International Convention on Information and Communication Technology, Electronics and Microelectronics (MIPRO).

[51] Raspberry Pi 3 Model B. https://www.raspberrypi.org/products/raspberry-pi-3-model-b/.

[52] Raspberry Pi 3 Model B+. https://www.raspberrypi.org/products/raspberry-pi-3-model-b-plus/.

[53] Aksentijević S., Krnjak D., Tijan E. (2015). Logistics environment awareness system prototype based on modular Internet of Things platform. Pomorstvo.

[54] Ismail L., Materwala H., Zeadally S. (2019). Lightweight Blockchain for Healthcare. IEEE Access.

[55] Ren Y., Leng Y., Zhu F., Wang J., Kim H.J. (2019). Data Storage Mechanism Based on Blockchain with Privacy Protection in Wireless Body Area Network. Sensors.

[56] Hang L., Kim D.H. (2019). Design and Implementation of an Integrated IoT Blockchain Platform for Sensing Data Integrity. Sensors (Basel).

[57] Uddin M.A., Stranieri A., Gondal I., Balasubramanian V. (2018). Continuous patient monitoring with a patient centric agent: A block architecture. IEEE Access.

[58] Shahid A.R., Pissinou N., Staier C., Kwan R. Sensor-Chain: A Lightweight Scalable Blockchain Framework for Internet of Things. Proceedings of the 2019 International Conference on Internet of Things (iThings) and IEEE Green Computing and Communications (GreenCom) and IEEE Cyber, Physical and Social Computing (CPSCom) and IEEE Smart Data (SmartData).

